# Community-Based Entomological Surveillance Reveals Urban Foci of Chagas Disease Vectors in Sobral, State of Ceará, Northeastern Brazil

**DOI:** 10.1371/journal.pone.0170278

**Published:** 2017-01-19

**Authors:** Cynara Carvalho Parente, Fernando S. M. Bezerra, Plutarco I. Parente, Raimundo V. Dias-Neto, Samanta C. C. Xavier, Alberto N. Ramos, Filipe A. Carvalho-Costa, Marli M. Lima

**Affiliations:** 1 Faculdade de Medicina, Universidade Federal do Ceará/Sobral, Sobral, Ceará, Brazil; 2 Faculdade de Farmácia, Odontologia e Enfermagem, Universidade Federal do Ceará, Fortaleza, Ceará, Brazil; 3 Centro de Controle de Zoonoses, Secretaria Municipal de Saúde de Sobral, Sobral, Ceará, Brazil; 4 Laboratório de Biologia de Tripanossomatídeos, Fundação Oswaldo Cruz, Fiocruz, Rio de Janeiro, Rio de Janeiro, Brazil; 5 Faculdade de Medicina, Universidade Federal do Ceará, Fortaleza, Ceará, Brazil; 6 Laboratório de Epidemiologia e Sistemática Molecular, Instituto Oswaldo Cruz, Fiocruz, Rio de Janeiro, Brazil; 7 Escritório Técnico Regional Fiocruz, Teresina, Piauí, Brazil; 8 Laboratório de Ecoepidemiologia da Doença de Chagas, Instituto Oswaldo Cruz, Fiocruz, Rio de Janeiro, Brazil; Universidade Federal de Minas Gerais, BRAZIL

## Abstract

**Background:**

The aim of this work was to explore the potential risk of vector-borne Chagas disease in urban districts in northeastern Brazil, by analyzing the spatiotemporal distributions and natural infection rates with *Trypanosoma cruzi* of triatomine species captured in recent years. The main motivation of this work was an acute human case of Chagas disease reported in 2008 in the municipality of Sobral.

**Methodology/principal findings:**

We analyzed data from community-based entomological surveillance carried out from 2010 to 2014. Triatomine natural *T*. *cruzi* infection was assessed by examination of insect feces by optical microscopy. Sites of triatomine capture were georeferenced through Google Earth and analyzed with ArcGIS. A total of 191 triatomines were collected, consisting of 82.2% *Triatoma pseudomaculata*, 7.9% *Rhodnius nasutus*, 5.8% *T*. *brasiliensis*, 3.7% *Panstrongylus lutzi*, and 0.5% *P*. *megistus*, with an overall natural infection index of 17.8%. Most infestations were reported in the districts of Dom José (36.2%), Padre Palhano (24.7%), and Alto do Cristo (10.6%). The overwhelming majority of insects (185/96.9%) were captured inside houses, and most insects tended to be collected in intermittent peaks. Moreover, captured triatomines tended to constitute colonies. The acute case reported in 2008 was found to be situated within a *T*. *pseudomaculata* hotspot.

**Conclusion:**

The triatomine collection events carried out by dwellers were aggregated in time and space into distinct foci, suggesting that insects are intermittently and artificially introduced into the city, possibly via accidental migration from their natural reservoirs. The relatively high *T*. *cruzi* infection rate indicates considerable circulation of the parasite in these areas, increasing the risk of vector-borne Chagas disease infection. These data suggest a need to strengthen epidemiological surveillance and integrate appropriate control actions targeting triatomines, *T*. *cruzi* reservoirs, and human populations. Our data also identify Chagas disease transmission as a hazard in urban areas of Sobral.

## Introduction

Bloodsucking insects of the subfamily Triatominae are distributed widely throughout the Americas and are also vectors of the protozoan *Trypanosoma cruzi*, the causative agent of Chagas disease. Several triatomine species are of great epidemiological importance due to their high susceptibility to *T*. *cruzi* infection as well as their ability to invade and colonize households, the combination of which increases the risk of Chagas disease transmission. Chagas disease was first described by the Brazilian scientist and physician Carlos Chagas at the beginning of the 20^th^ century [1]. This disease has remained a serious public health problem in Latin America, where about 5 to 7 million people are currently infected, resulting in approximately 12,000 deaths annually [2]. Chemical control of introduced/domiciled insect vectors, mainly *Triatoma infestans* [3], and progressive socioeconomic and housing improvement [4] have helped successfully interrupt vector-borne disease transmission in many regions. Thus, in the last few decades, most new cases in Brazil have corresponded to acute Chagas disease and have been associated with food-borne outbreaks. Such outbreaks have been a particular problem in northern Brazil (e.g. in the Amazon), where some juices have been contaminated with sylvatic triatomine feces containing *T*. *cruzi* [5,6,7]. In addition to acute infections, 1.9 to 4.6 million people are estimated to be chronically infected, approximately 1.0 to 2.4% of the Brazilian population, resulting in about 6,000 deaths annually [8]. Chagas disease was endemic in the state of Ceará in northeastern Brazil [9], transmitted primarily by native triatomines migrating from the wild to dwellings. These triatomines are able to recolonize peridomiciles and intradomiciles shortly after insecticide control measures have been implemented [10,11], posing difficult challenges for Chagas disease control.

Based on current stratification of Brazilian municipalities by various entomological, environmental, and demographic indicators, the city of Sobral is considered to be at high risk for vector-borne Chagas disease. Of particular importance, native triatomine species such as *T*. *brasiliensis* and *T*. *pseudomaculata* are often found inside houses, sometimes carrying *T*. *cruzi* infection [12]. In 2008, an autochthonous acute case of Chagas disease was reported in a neighborhood in Sobral, leading health authorities to conduct an entomological survey that identified 21 specimens of *T*. *pseudomaculata*, 19 of which were collected inside the index-patient household, 18 of which were infected with *T*. *cruzi* [12]. Thus, there is an urgent need to precisely map the spatiotemporal distribution of vectors in this urban area.

In this study, we aimed to explore potential risks of Chagas disease infection in the urban area of Sobral by characterizing the spatial and temporal capture trends of Chagas disease vectors in this area.

## Materials and Methods

### Description of the study area

**Fig 1:**
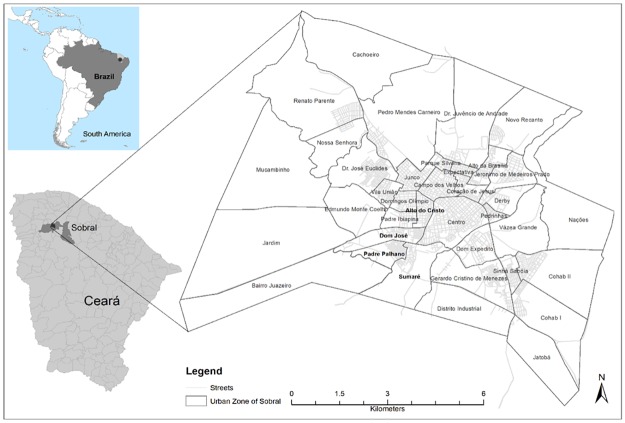
Location of the municipality of Sobral, Ceará State, northeastern Brazil. Collection sites were georeferenced with Google Earth (base map modified from IBGE (ftp://geoftp.ibge.gov.br/recortes para fins estatísticos/malha de setores censitários/censo2010/base de faces de logradouros).

**Fig 2:**
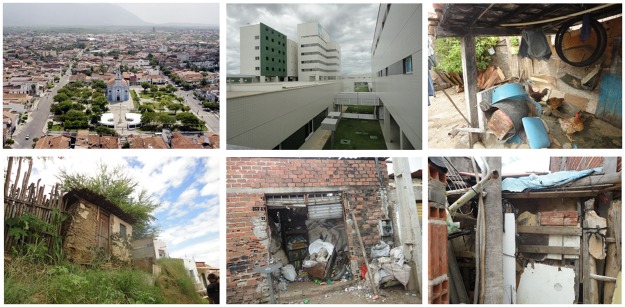
Landscapes of Sobral, Ceará, Brazil. A: Aerial view of the city. In the background one can observe the Serra da Meruoca, a region of preserved forest near the city; B: Modern buildings in the city center; C: Chickens and domestic devices piled in the peridomicile; D: Clay house with cracks, vulnerable to colonization by triatomines; E: Garbage accumulated in a shed, attractive for rodents; F: Waste accumulated in a yard, site potentially harboring triatomines.

Sobral (3° 41´06.39° S; 40° 20´52.87° E) is one of the largest cities in the state of Ceará and is an important commercial, industrial, and educational hub in the region (Fig 1). The municipality has a geographic area of 2,122.898 km^2^, a population of 193,134 inhabitants (population density of 86.67 habitants/km^2^), and 64 districts [Instituto Brasileiro de Geografia e Estatística (IBGE), 2015]. The majority of the population is densely concentrated in the urban districts of the heart of the municipality. Sobral is the largest economy in the inland of Ceará, the second most developed city in Ceará (after the capital, Fortaleza, according to the Human Development Index), and the third largest economy in the inland of the Brazilian Northeast. Paradoxically, the migration of peasants from rural localities to the city has produced substandard housing conditions in impoverished urban districts, mainly the districts of Padre Palhano, Dom José, Alto do Cristo, and Sumaré (Fig 2). The climate is hot and dry (semi-arid); annual temperatures range from 22.2–38.8◦C (altitude of 70 meters above sea level). There is a rainy season in the first semester, from January to May (average monthly rainfall in this period can reach as high as 173 mm). Rainfall reduces significantly in the second semester, with a monthly average of 4 mm from August to November. Since 2011 the seasonal rains have not fallen with their normal intensity, causing prolonged drought. The predominant vegetation is typical Caatinga, specifically, spinous deciduous forest.

### Community-based entomological surveillance

Data collected by the Zoonosis Control Center (ZCC), generated by the Triatomine Information Stations (PIT—acronym in Portuguese) in the urban area of Sobral from 2010 to 2014 were included in the analysis. As part of the National Control Program of Chagas Disease, PITs were established in formerly endemic Brazilian municipalities, with the objective of monitoring the presence of infected insects that can transmit Chagas disease. Typically, the station is situated at a site chosen by health surveillance authorities where members of the community can readily notify suspicious insects. From the PIT, the insects are transported to a reference laboratory, where they are identified at the species level. At the reference laboratory, triatomine natural *T*. *cruzi* infection is also verified by examination on a light optical microscope of fresh feces obtained by abdominal compression. Typically, PITs are installed in locations accessible to people living in particular geographic areas. The urban heart of Sobral harbors a single PIT in the Zoonosis Control Center, and all residents have access to it. Therefore, the regional PIT networks comprise a passive surveillance strategy of Chagas disease vectors and receive suspicious insects from members of the local community. After forwarding to a central level, local health authorities perform a detailed inspection of the household is carried out and insecticide is applied, if deemed necessary. Specifically, if triatomine colonization is found inside houses or in the peridomicile, pyrethroid insecticide spraying is performed by health authorities.

From the initial phase of chemical control of Chagas disease vectors in the 1980s, the population of endemic areas such as the Brazilian Northeast has been made aware of the need to maintain a high level of active community-based surveillance, since the possibility of reinvasion of the domestic environment by native triatomines is high. Therefore, during their activities, which include home visits, the surveillance agents of endemic diseases in Sobral encourage the residents to collect the insects they suspect to be triatomines and send them to the PIT.

The following variables were included in the analysis: triatomine species, sex, evolutionary stage, house address and location (intra or peridomicile) where the insect was found, date of capture (including month and year), and *T*. *cruzi* infection rates. The overall index of natural infection was calculated as the ratio of the number of infected triatomines to the number of examined triatomines. This study was approved by the Research Ethics Committee of Ceará Federal University (protocol number 35/2014).

### Geospatial analysis

The base map and streets were acquired from the Brazilian Institute of Geography and Statistics (IBGE, census 2010. Division of neighborhoods in the metropolitan region of Sobral was supplied by Geoprocessing Sector of the Sobral City Hall mapping. Google Earth^®^ was used to determine the coordinates of all triatomine collection sites. Coordinates were recorded in the WGS 84 Datum (World Geodetic System 1984) geodetic coordinate system. For exploration and modeling, the maps were analyzed for spatial point patterns by using the kernel method. A quartic analysis method was used to allow spatial variation in the point densities, evaluating only the first order effects.

## Results

**Fig 3:**
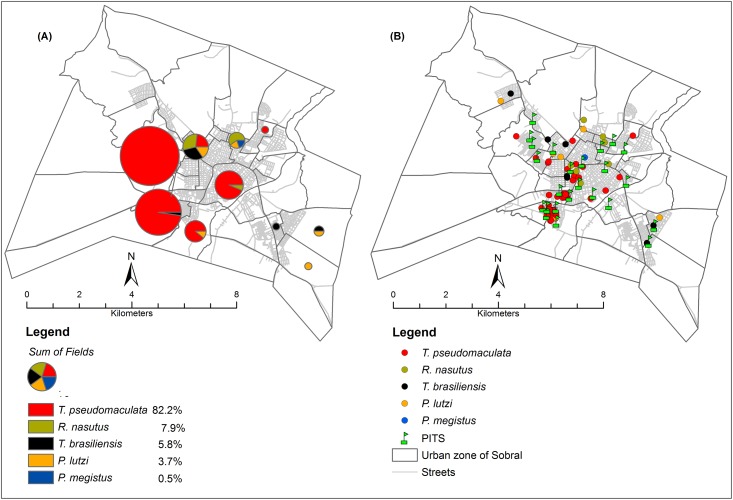
(A, B)—Mapping of the triatomine distribution in the urban area of Sobral. A—Relative distribution of the collected triatomine species: the chart sizes indicate the total number of the captured specimens, whereas pies displays the percentage of collected species. B—Spatial distribution of collected triatomine species related to PIT spots.

From 2010 to 2014, the residents of Sobral collected 191 triatomines, which were later laboratory-confirmed as the following species: *Triatoma pseudomaculata* (n = 157), *Rhodnius nasutus* (n = 15), *T*. *brasiliensis* (n = 11), *Panstrongylus lutzi* (n = 7), and *Panstrongylus megistus* (n = 1). The overall index of natural *T*. *cruzi* infection was 17.8%. The *T*. *cruzi* infection rates by insect species were as follows: *T*. *pseudomaculata* 17.8%, *R*. *nasutus* 13.3%, *T*. *brasiliensis* 9.1%, *P*. *lutzi* 28.6%, and *P*. *megistus* 0%. The insects were captured with similar frequencies in the dry (n = 97, 50.8%) and rainy (n = 94, 49.2%) seasons. Similarly, *T*. *cruzi* infection rates were similar in the dry (17 positive insects, 17.5%) and rainy (17 positive insects, 18.1%) seasons. Fig 3A and 3B shows the relationship between triatomine distribution and PIT locations where insects were collected in densely populated urban areas. *R*. *nasutus* was found mainly in the central and northern districts, while *P*. *lutzi* and *T*. *brasiliensis* were collected mainly in the central and northwestern areas. These geospatial analyses lead us to understand the extent to which vector distribution patterns are explained by actual infestation or community surveillance actions, so that meaningful lessons can be drawn from public health policies and interventions.

**Fig 4:**
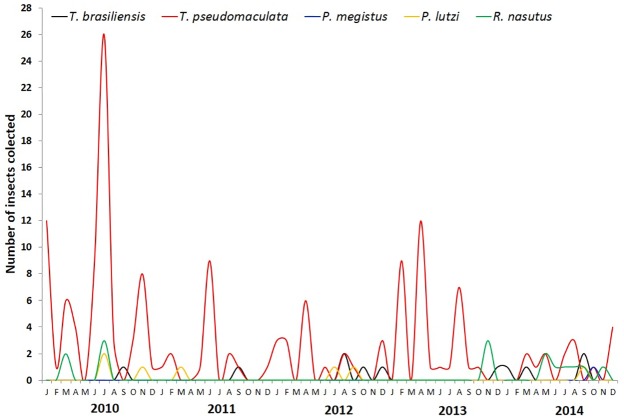
Numbers of triatomine insects found in the urban area of Sobral, Ceará, Brazil, from 2010–2014, according to species by month and year.

The vectors were reported in 16 out of the 64 districts under surveillance. Insects were collected most frequently in the Dom José (36.2%), Padre Palhano (24.7%), and Alto Cristo (10.6%) districts. Most insects (185/96.9%) were captured within domiciles, with only 6 specimens found in peridomiciles. Among the insects found in intradomiciles, 18.4% (n = 34) were *T*. *cruzi*-infected. Adult insects (both males and females) represented 74.9% of the captured insects, while nymphs represented 25.1%, indicating household colonization. Six (12.5%) of the 48 captured nymphs presented *T*. *cruzi* infection. Of 191 specimens collected, 114 (60%) were found with other triatomines at the time of collection. This co-infestation was found in 28 of the 144 houses infested, indicating that the captured triatomines were forming aggregate colonies. Insects were collected in 49 months of the 60-month surveillance period. A marked feature of their temporal distribution was that the insects tended to be collected in intermittent peaks. Specifically, the majority of triatomines (140, 73.3%) were captured in only 17 different months throughout the study period (17/49, 34.7%) (Fig 4).

**Fig 5:**
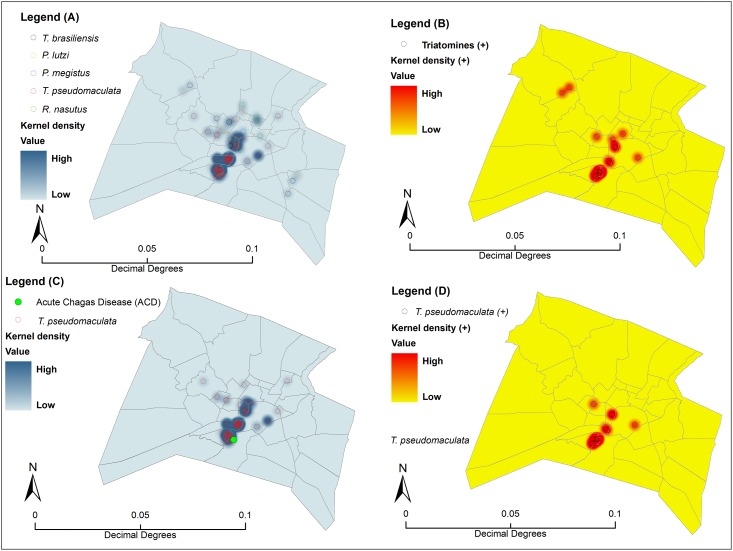
Map of the spatial distribution of the triatomines collected in the urban region of Sobral, Ceará, Brazil. The pictures framed in blue (A) represent the distribution mapping of triatomine species collected in Sobral residences (*Triatoma pseudomaculata*, *Rhodnius nasutus*, *T*. *brasiliensis*, *Panstrongylus lutzi*, and *P*. *megistus*); the pictures framed in yellow (B) represent *Trypanosoma cruzi* infection among the examined triatomines; the pictures framed in blue (C) represent the distribution of *T*. *pseudomaculata*; and the pictures framed in yellow (D) represent *T*. *cruzi* infection in *T*. *pseudomaculata*.

Geomapping of collection events indicated that the insects were clustered in the urban heart of Sobral (Fig 5A and 5C). The data surface density map also enabled the identification of potential transmission hotspots, which are related regions in which high numbers of *T*. *cruzi*-infected triatomines were collected (Fig 5B and 5D). The location of the acute case reported in 2008 appears to be geographically close to the hotspots of *T*. *pseudomaculata* captures (Fig 5C).

In addition to these findings, the PIT was alerted to the presence of a triatomine colony on the roof of a historic church in the downtown district that was being repaired by construction workers. The ZCC inspected the church roof and captured 15 *T*. *pseudomaculata* insects (six adults and eight nymphs, one of which was *T*. *cruzi*-infected), and one winged, uninfected *R*. *nasutus* insect.

## Discussion

This study demonstrates the presence of urban spotlights of triatomines, vectors of Chagas disease, in densely populated neighborhoods of a city in northeastern Brazil. This finding represents a detailed account showing the presence of these insects in an urban environment, since Chagas disease is typically considered to be endemic in rural settings. Moreover, in northeastern Brazil, Chagas disease is usually thought to be transmitted by insects with natural reservoirs that invade and colonize houses from the wild [13]. Therefore, Sobral seems to exhibit a unique eco-epidemiological scenario characterized by triatomine foci in informal urban settlements with substandard housing. These foci have the potential to produce acute Chagas disease cases, and represent a real risk to the population. This situation underscores interruption of vector-borne Chagas disease infection in this region as one of the main challenges of insecticide-based control policies. The triatomine collection events carried out by city dwellers were aggregated in both time and space, with insect collections restricted to only a few foci in the city. Moreover, *T*. *pseudomaculata* temporal peaks were detected, the largest of which was observed in 2010. This finding suggests that the insects are introduced into the city intermittently and artificially, and possibly undergo passive transport from their natural reservoirs (i.e., in the Caatinga biome that surrounds the city). Moreover, the acute Chagas disease case reported in 2008 was spatially related to these foci.

As discussed by Ramos Jr. and Carvalho [14], native triatomines are the main vectors of Chagas disease in large areas in the Brazilian Northeast. Several species are native to Ceará, including *T*. *brasiliensis* and *T*. *pseudomaculata* [15], presently considered two of the most notorious autochthonous species in Brazil [16]. Of the 89,349 Chagas-related deaths recorded in Brazil between 1996 and 2013, 16,726 (almost 20%) occurred in northeastern Brazil (DataSUS 2015, http://tabnet.datasus.gov.br). This statistic highlights the competence of native triatomine species to transmit *T*. *cruzi* to humans.

An interesting question is which natural environments continually supply these insects to homes in different neighborhoods of Sobral. A piece of evidence that may help answer this question is our finding that *T*. *pseudomaculata* accounted for over 80% of the captured specimens. *T*. *pseudomaculata* is predominantly an arboreal species whose primary habitat consists of tree trunks. Insects of this species take shelter beneath the bark of trees [particularly *Mimosa tenuiflora* (Jurema Preta), which is extremely common in the Caatinga vegetation] and feed on bird blood. However, *T*. *pseudomaculata* can also be found in many other tree species, including *Caesalpinia pyramidalis* (Catingueira), *Spondias tuberosa* (Umbuzeiro), *Commiphora leptophloeos* (Imburana), *Schinopsis brasiliensis* (Barauna), *Sideroxylon obtusifolium* (Quixabeira), *Anadenthera* spp (Angico), *Lithraea molleoides* (Aroeira), *Aspidosperma pyrifolium* (Pereiro), and Faveleira (*Cnidoscolus quercifolius)* [17]. One possibility is that the deforestation associated with urban expansion has resulted in the destruction of natural habitats, which can reduce or eliminate the insect food sources, resulting in household invasion. In support of this hypothesis, the extraction of native wood for the production of charcoal and firewood is an important economic activity in Sobral. This extraction produced 185 tons of charcoal and 112,700 cubic meters of firewood in 2014 (IBGE 2016; http://www.cidades.ibge.gov.br/xtras/perfil.php?lang=&codmun=231290). This statistic exemplifies the impact of the Caatinga biome deforestation.

The capacity of *T*. *pseudomaculata* to fly towards light is well-known. Moreover, although triatomines usually move by crawling, flight dispersal is essential for the colonization of new habitats [18,19]. In addition, it was also demonstrated that *T*. *pseudomaculata* is highly adaptable to different habitats and can occupy substrates distinct from the sylvatic environment [18,20]. Passive spread of *T*. *pseudomaculata* from the wild into the urban area is also a possibility. In support of this hypothesis, the transport of firewood obtained from infested trees and the subsequent storage of this firewood in peridomicile environments have been shown to introduce nymphal and adult triatomine species, such as *T*. *pseudomaculata* [21] and *T*. *brasiliensis* [22], into houses. Although in the natural environment *T*. *pseudomaculata* prefers to feed on bird blood, when established in a domestic environment it can feed on rodents (such as rats) and dogs [23,24] and can thus become infected with *T*. *cruzi*. We found that almost one-fifth of the *T*. *pseudomaculata* specimens collected in the Sobral urban districts were *T*. *cruzi*-positive, an alarmingly high rate.

Another species often found in Sobral residences is *R*. *nasutus*. The natural habitats of this species are palm trees, such as *Copernicia prunifera*, which are abundant in the region [25,26,27]. However, the habitat of *R*. *nasutus* has been shown to be relatively flexible, with specimens also found in *Licania rigida* trees (Oiticica) [28]. We propose that, like *T*. *pseudomaculata*, *R*. *nasutus* was attracted to houses in Sobral in search of food due to the environmental changes caused by urban expansion. The *T*. *cruzi* positivity rate of *R*. *nasutus* was 6%, suggesting that these insects may also be feeding on mammalian Chagas disease reservoirs in the city. Other species such as *T*. *brasiliensis*, *P*. *lutzi*, and *P*. *megistus* were also observed in Sobral, some of which were also *T*. *cruzi*-positive. These insects may also have been attracted to houses from their environmental reservoirs.

## Conclusion

Sobral exhibits a unique eco-epidemiological situation, in which Chagas disease vectors are continuously present inside houses in different neighborhoods, thereby creating constant contact between humans and triatomines. This close contact presumably enables the emergence of Chagas disease cases. However, the frequency of this emergence is unknown, since acute *T*. *cruzi* infections are usually asymptomatic. A comprehensive investigation is urgently needed to determine which urban mammals are infected with *T*. *cruzi* and the seroprevalence of Chagas disease in the residents of this area. Community-based entomological surveillance must become a strategic component of Chagas disease control in this area, and will be strengthened by close involvement of major stakeholders, such as the residents themselves and the Sobral health authorities who should promote policies of assistance, surveillance and vector control. This cooperation between the population and local authorities will contribute decisively to avoid the emergence of new cases of the endemia.
